# Advances towards the complete *in vitro* life cycle of *Toxoplasma gondii*

**DOI:** 10.12703/r/12-1

**Published:** 2023-02-13

**Authors:** David Warschkau, Frank Seeber

**Affiliations:** 1 FG16: Mycotic and Parasitic Agents and Mycobacteria, Robert Koch-Institut, Berlin, Germany

**Keywords:** *Toxoplasma gondii*, toxoplasmosis, life cycle, in vitro model, organoids, bradyzoites, oocysts

## Abstract

The full life cycle of *Toxoplasma gondii* cannot be recapitulated *in vitro*, and access to certain stages, such as mature tissue cysts (bradyzoites) and oocysts (sporozoites), traditionally requires animal experiments. This has greatly hindered the study of the biology of these morphologically and metabolically distinct stages, which are essential for the infection of humans and animals. However, several breakthrough advances have been made in recent years towards obtaining these life stages *in vitro*, such as the discovery of several molecular factors that induce differentiation and commitment to the sexual cycle, and different culture methods that use, for example, myotubes and intestinal organoids to obtain mature bradyzoites and different sexual stages of the parasite. We review these novel tools and approaches, highlight their limitations and challenges, and discuss what research questions can already be answered with these models. We finally identify future routes for recapitulating the entire sexual cycle *in vitro*.

## Introduction

Apicomplexan pathogens have evolved complex life cycles featuring finely balanced persistent and replicative stages, including both asexually proliferating forms and sexual recombination, to ensure optimal spread of the parasite. Experimental access to these stages is paramount to study the pathogenic mechanisms and to develop urgently needed therapies and vaccines. The most widespread member of the Apicomplexa is *Toxoplasma gondii*, causative agent of toxoplasmosis, which has a broad host range and can infect any warm-blooded animal and humans (for review see 1,2). An estimated 25% of the world’s population is infected with the parasite, with large local variations^3^. While *T. gondii* infections are generally controlled in immunocompetent individuals, the parasite establishes chronic, lifelong infections that pose the risk of reactivation and severe disease when the immune system becomes compromised. There is currently no approved therapy to clear chronic infections^4^.

**Figure 1.**
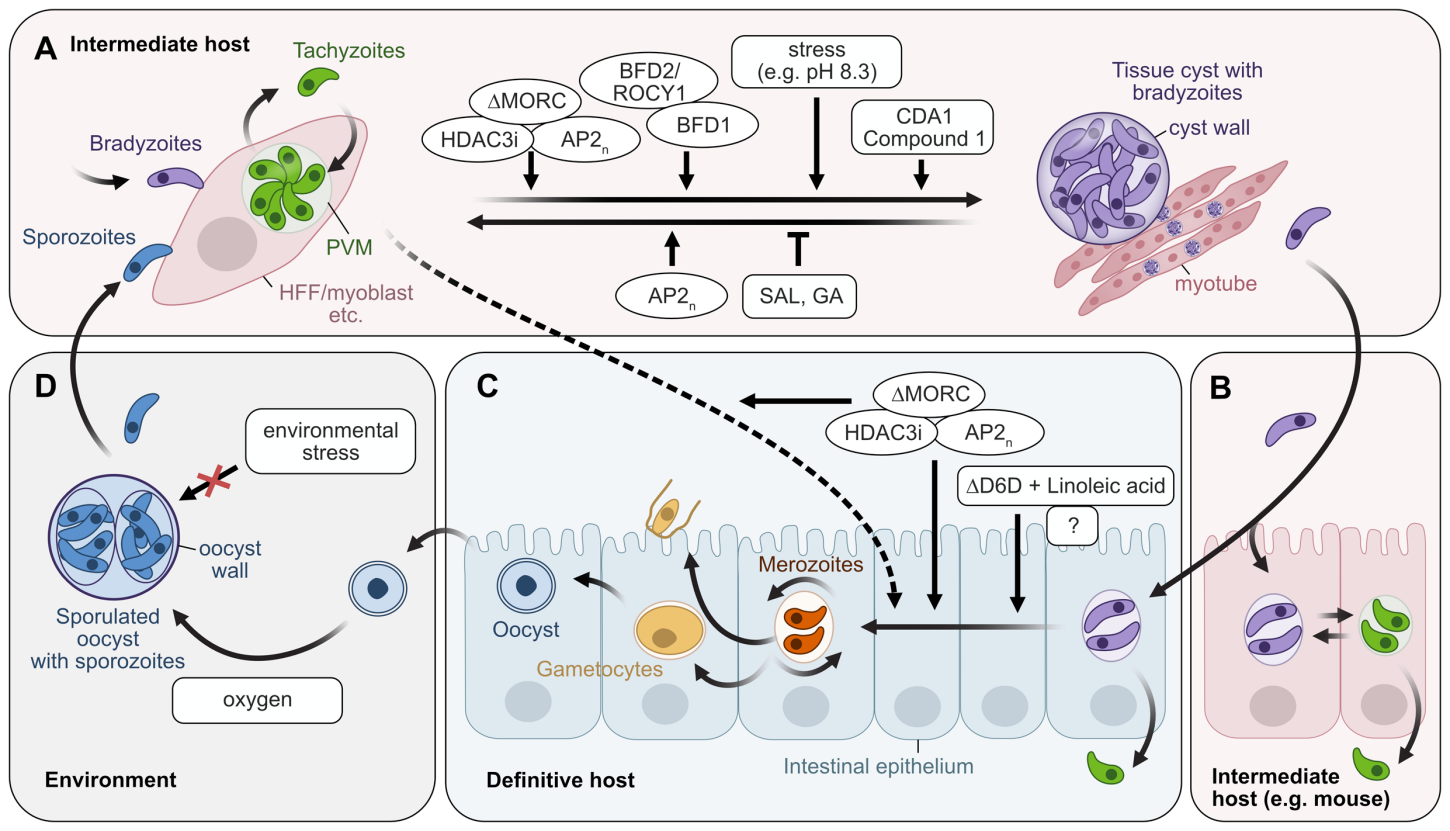
Life cycle of *T. gondii* and factors with known contribution to stage conversion. (**A**) Upon invasion of any nucleated host cell tachyzoites, bradyzoites or sporozoites form a parasitophorous vacuole surrounded by a membrane (PVM) and eventually multiply and disseminate as tachyzoites. *In vitro*, human foreskin fibroblasts are most frequently used for tachyzoite cultivation. The chronic bradyzoite stage forms in tissue cysts in the eye, brain, and muscle tissue. *In vitro*, bradyzoite formation is induced by various stresses (e.g., alkaline cell culture medium pH 8.3; see text for additional stressors). Overexpression of *BFD1, BFD2/ROCY1*, deletion of MORC or inhibition of HDAC3 induce bradyzoite formation *in vitro*. Bradyzoites can reactivate to tachyzoites, which is attenuated by elF2⍺ dephosphorylation inhibitors salubrinal (SAL) and guanabenz (GA)^5^. Several transcription factors of the Apetala 2 (AP2_n_) family are involved at various steps. (**B**) After ingestion of tissue cysts, bradyzoites invade the intestinal epithelium of intermediate hosts and differentiate to tachyzoites. These pass the intestinal epithelium and disseminate throughout the host. (**C**) Only in the intestinal epithelium of felines, where linoleic acid content is high due to a natural delta-6-desaturase (D6D) deficiency, can bradyzoites commence with the sexual developmental pathway. Merozoites replicate asexually, commit to a sexual fate, give rise to male and female gametocytes, eventually leading to oocyst formation. Further currently unknown factors (“?”) might be required. Similar conditions can be mimicked in mouse intestinal organoid-derived cells when D6D activity is inhibited and, in addition, linoleic acid is supplemented^6^. Inhibition of MORC or HDAC3 induce sexual stage-specific gene expression in tachyzoites (dashed line). (**D**) Eventually, oocysts are shed with the feline’s feces and sporulate in the environment under aerobic conditions. Oocysts are resistant to environmental stressors such as ultraviolet irradiation, salinity, high and low temperatures, and desiccation.

Transmission occurs mostly through consumption of raw or undercooked meat containing tissue cysts or by ingestion of sporulated oocysts, the environmental stage of *T. gondii*, present in contaminated water, soil, or unwashed produce (Figure 1A). Following infection, the transmissive parasite stages, bradyzoites and sporozoites, invade the intestinal epithelium, convert into the fast-replicating tachyzoite stage, and disseminate throughout the host (Figure 1B, C). Parasites then differentiate into bradyzoites within tissue cysts that are found primarily in the eye, brain and muscle tissue (Figure 1A). In addition to this asexual replication, sexual recombination can take place in felines, the definitive hosts (Figure 1C)^1^. The sporozoite-containing oocysts formed during this process are shed into the environment with the feces and can withstand harsh environmental stresses such as ultraviolet irradiation, salinity, high and low temperatures, and desiccation, allowing them to remain infectious for months and even years (Figure 1D)^7^.

While tachyzoites can be readily cultured *in vitro*, research of persistent infections and sexual development, as well as access to mature bradyzoites and oocysts, have traditionally depended on animal infections^8^. While many aspects of the disease can only be recapitulated in animal models, ethical concerns, especially the use of cat infections for oocyst generation, have resulted in increased efforts to establish alternative *in vitro* systems where appropriate. Cell culture models would offer additional advantages, such as greater control over experimental parameters, reproducibility, and scalability. They also allow the use of methods that are difficult to perform *in vivo*, for example, live-cell imaging or metabolomic studies, which ask for very specific conditions. It has resulted in remarkable progress in recent years, which is highlighted in this review. Our main focus will be on cell culture systems and *in vitro* methods to produce life stages in sufficiently high yield to serve as alternative sources for animal-derived parasite forms. It is noteworthy that the development of these models has led to increased understanding of the mechanisms of bradyzoite formation and sexual development itself.

## Culture models for tachyzoites

**Figure 2.**
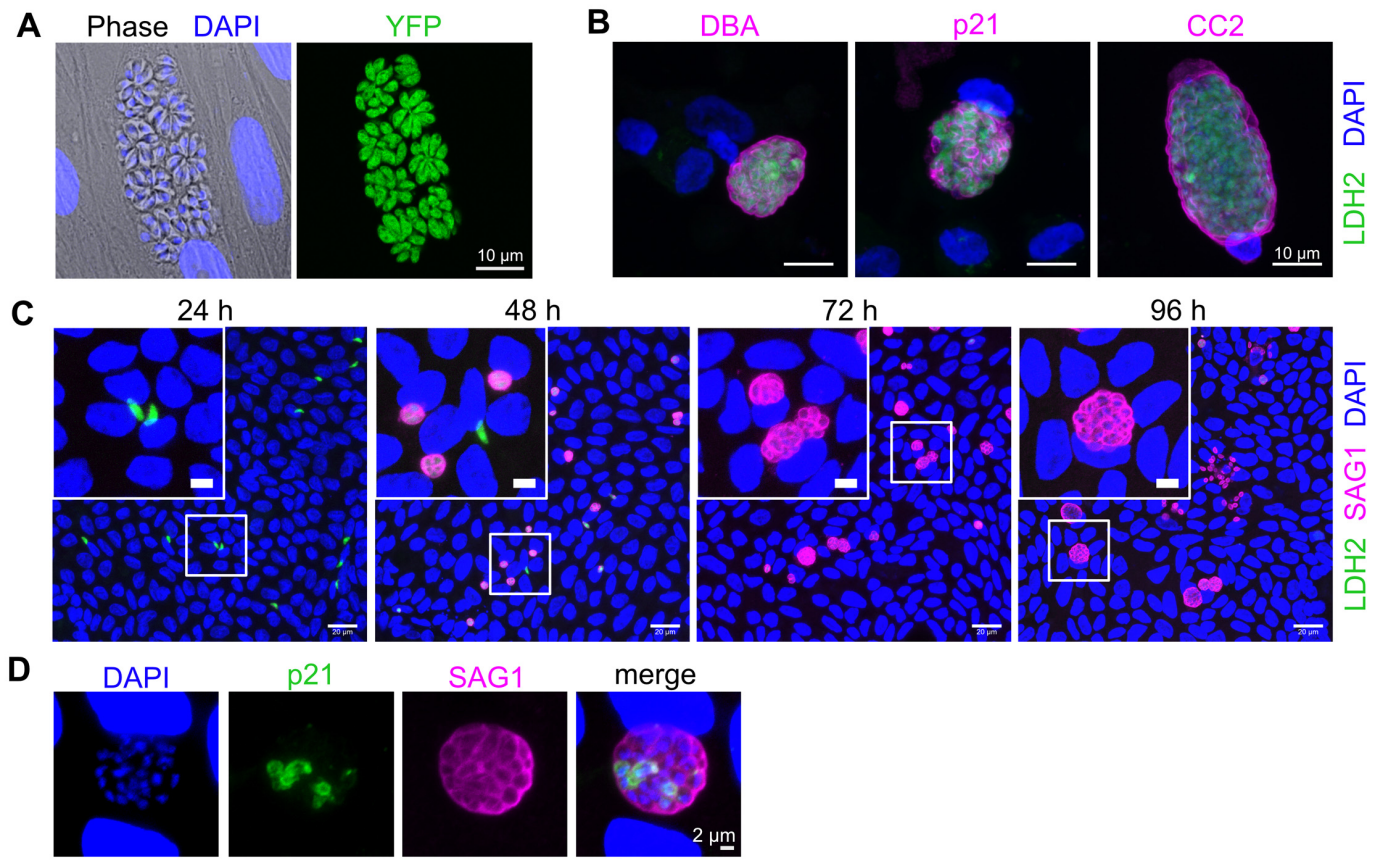
Examples of cell culture systems for *in vitro* growth of different *T. gondii* stages. (**A**) Proliferating tachyzoites can be propagated and maintained in human foreskin fibroblast cultures. Immunofluorescence images show GT1 strain parasites expressing YFP (green) in the cytoplasm, forming typical rosette assemblies in the parasitophorous vacuole (PV) within the host cell. Host and parasite nuclei are stained with DAPI in blue. (**B**) *In vitro* tissue cysts in human myotube cell cultures 28 days post infection with Pru strain parasites expressing GFP under the control of bradyzoite-specific LDH2 promoter^30^. Immunofluorescence images depict bradyzoite markers CST1, p21, and CC2, decorated by DBA or specific antibodies (for details see text). (**C**) Human intestinal organoid-derived monolayers (ODMs) were generated as described in 31 and were infected with *in vitro*-derived Pru bradyzoites from myotube cultures, as shown in **B**. Projections of immunofluorescence images of a z-stack, showing that the bradyzoites are able to invade the intestinal epithelial cells, replicate, and differentiate into tachyzoites, as indicated by SAG1 expression over time (magenta). DAPI-stained nuclei in blue. Scale bar = 20 µm. Inserts show an enlarged view of boxed areas with scale bar = 5 µm. (**D**) Example of a PV 96 h after bradyzoite infection of intestinal ODMs as in **C**, harboring individual parasites expressing the late bradyzoite marker p21 (green) among cells expressing only SAG1 (magenta). It most likely reflects tachyzoite-to-bradyzoite reconversion.

Given that *T. gondii* is able to infect virtually any nucleated cell and has such a broad host spectrum, it is not surprising that the fast-replicating tachyzoite stage of *T. gondii* can be readily maintained *in vitro* in almost any nucleated cell of warm-blooded animals (for review see 9). Indeed, the first report of cultivating *T. gondii* in avian embryos and cell culture dates back to 1929^10^. A decade later, the RH strain was isolated^11^ and is still the primary strain used for experiments in many labs. But it was not until the 1950s that the documented *in vitro* culturing of *T. gondii* began to increase considerably^12^. Today, for ease of use, contact-inhibited primary human foreskin fibroblasts (HFF) are most commonly used for propagation and maintenance of *T. gondii* tachyzoites, which are simply passed from one culture flask to the next (lytic cycle) (Figure 2A). Little efforts have been invested in recent years to improve tachyzoite culture systems. A 3D culture system has been recently described, in which infected VERO cells were subsequently embedded in a collagen matrix, thereby allowing the parasite-containing vacuole to expand in all three dimensions^13^. However, practical issues with this approach limit its usefulness compared to conventional 2D cultures. The apparent non-selectivity of host cells by tachyzoites is in contrast to the preference of bradyzoites for non-dividing neurons and muscle cells and the commitment to the sexual cycle exclusively in feline intestinal cells. 

Importantly, and known for decades, continuous *in vitro* cultivation in the lab leads to enhanced host-independent virulence features like accelerated lytic growth, but the exact reasons for this were ill-defined^14–16^. A recently described “evolve and resequencing” method has now shown that these changes are likely the result of transcriptional reprogramming driven by the extracellular milieu that tachyzoites experience *in vitro* during the lytic cycle^17^. Still, tachyzoite cell culture models have been invaluable to our understanding of *T. gondii* biology, also making it a model for other Apicomplexa, which are less easy to cultivate.

## Culture models for bradyzoites

Bradyzoites are considered a quasi-dormant stage as they show reduced proliferation compared to tachyzoites, although some growth and replication within cysts occurs^18^. This quasi-dormancy is presumably a reason why bradyzoites do not respond to current therapies like pyrimethamine-sulfadiazine, which are effective against acute infections by targeting actively replicating forms^4^. The metabolism in bradyzoites is remodeled, likely to cope with nutrient limitations, which may be another factor explaining the tolerance to antiparasitic treatments^19,20^.

## Generation of bradyzoites *in vivo*

For experimental purposes, bradyzoite formation can be initiated *in vivo* by infecting animals, most commonly mice. The host genetic background and parasite strain can have a major impact on the course of infection and the establishment of chronicity^9^. Virulent type I strains grow rapidly in mice and are less capable to transform into bradyzoites than slow-growing type II and III strains^21,22^. Therefore, mainly type II strains like ME49 are used for cyst production in mice^23,24^.

Mice are orally infected by feeding tissue cysts from prior infections (usually isolated from brains of infected mice); with oocysts from cat feces, or by intraperitoneal (ip) injection of tachyzoites^9^. Importantly, it is long known ‒ but not appreciated by all ‒ that the route of infection, in particular the use of tachyzoites for ip instead of bradyzoites for oral infection, can greatly influence disease outcomes in mice^25–28^. This is highlighted by a recent study which compared the course of tissue cyst infection (i.e. no tachyzoites were used), delivered either orally or via ip injection, in the C57BL/6J mouse model^29^. The authors concluded that ip-infected mice showed more severe signs of disease, faster parasite dissemination and an altered immune response with regard to proinflammatory cytokines, which was observable throughout the entire infection. Moreover, differences in long-term microbial dysbiosis and in IFNγ production in the brain in the chronic phase could be seen. It is conceivable that similar or even greater differences might be observed by the use of ip injection of tachyzoites, which are much simpler to obtain and could be a reason for their frequent use. Consequently, a method for *in vitro* cultivation of bradyzoites that functionally resemble those obtained from infected animals would be advantageous for unavoidable mouse experiments, notwithstanding the ethical issues.

## *In vitro* bradyzoite differentiation reaches the next level

Spontaneous bradyzoite differentiation has been observed as early as the 1960s in numerous cells and cell lines, for example HeLa cells, fibroblasts, astrocytes, neurons, and muscle cells (reviewed in 32,33), although the percentage of cyst formation has rarely been reported or was very low. A notable exception was a Brazilian isolate (EGS), which after four days of culture in LLC-MK2 epithelial kidney cells showed more than 70% of cyst-like structures positive for early bradyzoite markers^34^. A later report^35^ attributed this ‘spontaneous’ behavior to a mutated transcription factor (AP2IV-4; see also below) known to be involved in the tachyzoite ⇔ bradyzoite switch^36^.

A variety of differentiation-inducing stresses for *in vitro* cultures have been sought for and identified. They have been described in several recent reviews^37–39^. Arguably, the most frequently used trigger is cell culture medium with an alkaline pH (8.3). Other factors include heat shock (43°C^40^), nutrient starvation (arginine^41^, pyrimidine via CO_2_ depletion^42^), deprivation of low-density lipoprotein-derived cholesterol^43^, and metabolic inhibitors (e.g. oligomycin^44^). In addition, the cell cycle state of the host cell influences bradyzoite formation. For example, increased expression of human cell division autoantigen-1 (CDA1), which can be upregulated by a trisubstituted pyrrole termed ‘Compound 1’, has been shown to trigger bradyzoite development^45^. Moreover, bradyzoite formation is observed in cell cycle-arrested host cells like terminally differentiated myotubes, but not in their proliferating myo-blast form^33,46^. Notably, although the *in vitro* cysts obtained using these triggers resemble *in vivo* cysts in many respects, for example in the formation of a cyst wall that can be stained with *Dolichos biflorus* agglutinin (DBA) (Figure 2B) and in the expression of major bradyzoite antigens (e.g. BAG1), several differences have been reported^47^. One study compared transcriptomes of 4-day-old *in vitro* with 21-day-old *in vivo* bradyzoites and identified differential expression of numerous genes^47^. Nevertheless, the use of (alkaline) stress to trigger bradyzoite differentiation is easy to establish in a laboratory and provides key features of early bradyzoites. This is reflected in numerous studies, including recent ones, in which this method has been employed to examine, for instance, the role of Ca^2+^ in bradyzoite egress from cysts^48^ or the role of pantothenate biosynthesis in the two parasite stages^49^.

The physiological triggers in the natural host are less well known, yet we are beginning to understand how *T. gondii* and the closely related species *Hammondia hammondi* and *Neospora caninum* are able to sense environmental stress signals by comparing the different “inducers” between these species^50^ (reviewed in 39). One important mechanism is an integrated stress response involving eukaryotic translation initiation factor 2 (eIF2) kinases that respond to stresses by phosphorylating TgIF2. This dampens global translation and promotes preferential translation of mRNA involved in stress remediation^37,51^.

Stage conversion to bradyzoites is accompanied by differential gene expression involving various factors of transcriptional regulation that can also be exploited as tools to turn on differentiation. The Myb-like transcription factor ‘Bradyzoite Formation Deficient 1’ (BFD1) was recently identified in a genome-wide CRISPR/Cas9 screen as a central component of this regulatory network^52^. BFD1 binds to transcriptional start sites of many genes upregulated in bradyzoites and is both sufficient and necessary to drive tachyzoite-to-bradyzoite conversion^52^. This is in contrast to another set of transcription factors, proteins of the Apetala 2 (AP2) family, which are involved in many developmental processes in *T. gondii* (reviewed in 53; see also below). However, none of those individual factors has such a broad regulatory effect on bradyzoite differentiation as BFD1^52^. BFD1 was therefore termed “the master regulator”^52^. Following this seminal work, the regulatory network of BFD1 has been studied in more detail, and two laboratories have now independently provided evidence for a zinc finger motif-containing protein (ToxoDB ID TGME49_311100/TGVEG_311100) as a critical secondary effector of BFD1, which they named ‘BFD2’^54^ or ‘Regulator of Cystogenesis 1’ (ROCY1)^55^, respectively. A positive feedback loop was proposed, in which the novel factor promotes the translation of *BFD1* transcripts under stress conditions and which in turn further increases transcription of *BFD2/ROCY1*, thereby reinforcing the fate commitment^54^. Conditional transgenic overexpression of *BFD1* or *BFD2/ROCY1* could therefore be another valuable experimental tool to induce bradyzoite differentiation, even in the absence of stress. However, for how long such parasite cultures are stable over time (beyond a few days) in HFFs, i.e., not being destroyed by replicating, non-differentiated tachyzoites in the system, has not been reported. From an experimental point of view, this developmental plasticity (tachyzoite ⇔ bradyzoite) has practical consequences and always requires attention (see below).

### Long-term *in vitro* culture of mature cysts

Since it is long known that *in vivo* tissue cysts mature over time, reflected by thickening of the cyst wall and other changes in morphological features^56^, *in vitro* cysts that have only been cultured for a short period of days cannot be regarded as fully mature. Advances in cell culture systems and protocols have recently allowed prolonged cultivation times and thereby the formation of mature tissue cysts in primary rat brain cells^57^ and immortalized human muscle cells^19^. These two studies suggest that long-term *in vitro* cultivation and maturation of tissue cysts apparently succeeds particularly well in cell types for which a tropism exists in the natural host, and which are terminally differentiated. This is consistent with previous results that had shown that infection of these cell types results in a high percentage of spontaneous differentiation (reviewed in 20).

Using a complex cell culture model consisting of several cell types found in the brain of newborn rats (including not only neurons but also astrocytes, glial cells and oligodendrocytes), Mouveaux *et al*. reasoned that the presence of these cell types would provide metabolic support for neurons^57^. Tissue cysts formed in infected cells were stable for at least 14 days and were orally infectious for mice. While this system is very promising to study aspects related to brain infections by *T. gondii*, it relies on the use of newborn animals and yields only limited amounts of cysts per rat brain (4x10^5^ cysts). An alternative for such neurological host-parasite systems might be the recently described generation of human stem cell-derived brain neurospheres and cerebral organoids that also result in spontaneous bradyzoite generation^58,59^. However, quantitative data like cysts numbers etc. were not reported. Primary mouse neonatal astrocytes have recently also been used to study bradyzoite recrudescence, i.e. the switch back to tachyzoites, resembling *in vivo* reactivation^24^. In contrast to fibroblasts, a second round of recrudescence and bradyzoite-to-bradyzoite replication has been observed in these host cells. However, the continuous use of this model required repeated infection of mice and re-isolation of tissue cysts^24^. Thus, in contrast to the model described below, this *in vitro* system is not primarily intended for the production of large amounts of bradyzoites.

Christiansen *et al*. have taken another route and infected differentiated, multinucleated human myotubes derived from an immortalized myoblast cell line (KD3^60^) and obtained tissue cysts that were stable for at least 35 days (Figure 2B)^19^. They exhibited gradually increasing tolerance to antiparasitic agents, resistance to pepsin digestion (both increased with cyst age) and were infectious to mice by the oral route, thus resembling *in vivo* cysts. Surprisingly, the fast-growing *T. gondii* type I strain RH also formed bradyzoites to a large extent, although in other systems it usually fails to do so. Comparing the two culture systems in terms of animals involved, ease of handling and output of cysts, the myotube system has some advantages: it depends only on established cell cultures and is thus also scalable for metabolomic analyses and genetic screens (10^6^ cysts/150 cm^2^ cell monolayer)^19^. Other immortalized human myoblast cell lines have been described and are commercially available^61^, and preliminary experiments indicate that they perform similarly to KD3 cells (unpublished results). In the context of the 3R principles of use of animals (replacement, reduction, and refinement), model systems like these could replace mice as sources for mature tissue cysts in the future, especially for drug screens or experiments requiring *ex vivo* bradyzoites. However, these models should be optimized in terms of handling effort and the reduced use of animal-derived media components^62^ and expensive growth factors^63^.

### Bradyzoite-specific molecular and ultrastructural markers

The quality of cell culture alternatives for the production of tissue cysts is governed in particular by the ability to generate mature cysts. However, the definition of “mature cysts” is not easy. Tissue cysts have been reported to form in the brains of mice between 5 and 6 days after ingestion of bradyzoites^64^. However, these tissue cysts continue to develop and gradually change their morphology, which has been demonstrated by electron microscopy^56,65^. During this process, they grow from small cysts containing as few as two bradyzoites to structures containing several hundred bradyzoites^32^. Increased numbers of micronemes and amylopectin granules have been detected. By four weeks after infection, the disappearance of organelle membranes and the spillage of bradyzoite cytoplasmic content into the cyst matrix has been observed. These cysts were described to contain a mixture of intact and degenerating parasites^65^. Ferguson and Hutchison report that tissue cysts remain relatively stable for many months *in vivo*^56^.

The development of tissue cysts is also accompanied by transcriptional changes that allow monitoring of the degree of differentiation^47,66^. Common bradyzoite markers include CST1, a cyst-wall glycoprotein that can be stained by *Dolichos biflorus* agglutinin (DBA)^67^, and the cytoplasmic small heat shock protein BAG1^68,69^. Both appear as early as day one following stress induction *in vitro*^67–69^. Lactate dehydrogenase 2 (LDH2) expression has been described at 48 h after alkaline stress^70^. The p21 antigen (TGME49_238440^71^) seems to be a marker for more mature bradyzoites as it has been reported to be absent in bradyzoites *in vitro* for at least 96 h after alkaline treatment^40^. Although these markers can provide a general indication of the degree of differentiation, they are not ideal to reliably assess the maturity of tissue cysts. A recent analysis of RNA sequencing data provided new molecular markers of mature bradyzoites that distinguish them from alkaline-stressed ME49 parasites^24^. It allowed the authors to identify genes that are expressed at high levels in mature bradyzoites but are at lower levels in alkaline-stressed bradyzoites, indicating the degree of maturity. Lastly, the resistance of bradyzoites to gastric pepsin digestion, still regarded as an important difference between tachyzoites and bradyzoites, is not entirely given, at least when tested in conjunction with highly sensitive experimental *in vivo* infections^72^.

The maturity of tissue cysts may affect experimental outcomes and should be considered when designing experiments. For example, while three-day old *in vitro* cysts can already withstand short exposure to low doses of pyrimethamine^73^, the resistance to anti-parasitics gradually increases as tissue cysts mature^19^. Moreover, mature bradyzoites may differ with respect to the glycan-rich cyst wall (that becomes thicker in mature cysts) and the metabolically quiescent state. Consequently, the use of *in vitro*-generated bradyzoites that express common bradyzoite markers but are only several days old instead of mature tissue cysts can significantly influence the conclusion of experimental results, for instance in drug development targeting bradyzoites. From a translational perspective, it should also be considered that even tissue cysts isolated from mice may not be fully comparable to those found in chronically infected humans or other species, not only because of different genetic background, but also because tissue cysts might persist for several decades in the tissues of infected individuals.

## Towards the generation of *in vitro* oocysts

Bradyzoites are unique in that they can either revert to the tachyzoite stage or enter the sexual developmental pathway by developing into merozoites that expand asexually, progressing through five merozoite stages (A-E), and eventually form gametes that mate and develop into oocysts^74^ (Figure 1C). Commitment to sexual stages and further development is accompanied by profound transcriptomic changes, the analysis of which has greatly contributed to our understanding of the regulation of sexual development^75,76^ and of the makeup of oocysts and sporozoites^77,78^. However, the precise molecular characterization of the individual merozoite types as well as micro- and macrogametes has not yet been possible because these investigations rely on infected cats, which only a few labs use and which is problematic for ethical reasons. Detailed knowledge is also hampered by the absence of methods to enrich and isolate sexually committed merozoites and separate gametocytes, as well as the lack of scalable *in vitro* models for the generation of sexual stages (for review see 79).

### Interfering with epigenetic regulation in tachyzoites causes the expression of merozoite and sporozoite genes

Remodelers of histone acetylation as regulators of gene expression have been extensively studied in *T. gondii* because they are possible targets for therapeutic interventions (for review see 80). Differential expression of stage-specific genes was observed when treating tachyzoites with sublethal doses of inhibitors of histone deacetylase 3 (HDAC3i) such as apicidin^81–83^, FR235222^81,84,85^, and recently MC1742^86^. Although the gene sets induced by these compounds are a mixture of distinct life cycle stages, these inhibitors may serve as a tool to obtain hybrids of sexual stages in cell culture. For instance, a sexual stage-specific mRNA expressed upon inhibitor treatment could be useful for convenient development and validation of primers used for reverse transcription quantitative PCR, without a need to obtain or isolate the respective parasite stage. As a surrogate for oocyst/sporozoite proteins, HDAC3-inhibited tachyzoites might be explored to identify appropriate proteins for vaccination^87^ or as a source for oocyst-specific diagnostic antigens^88^. The inhibitors could conceivably also be of use for studying protein-protein interactions specific to sexual stages and oocysts, provided that proper controls are implemented. Available RNA-seq data may help to assess the relevance of these models, keeping in mind that RNA expression does not necessarily equal protein translation^86^.

A breakthrough in our understanding of *T. gondii* stage transition was the identification of a central epigenetic regulator of sexual stage commitment in *T. gondii,* the microrchidia (MORC) protein, which is part of a repressor complex that controls the expression of stage-specific genes^84^. Most of them are enteroepithelial sexual stage (EES)-specific genes and, to a lesser extent, sporozoite- and bradyzoite-specific genes. MORC interacts directly with regulatory elements but also *via* recruiting and regulating additional factors such as AP2 transcription factors and HDAC3 (for review see 89). MORC was also discussed as a potential signaling hub able to process external cues^89^. These could be, for example, metabolic changes due to the intestinal environment. Considering the broad range of primary and secondary AP2 factors that are associated with and regulated by MORC, it seems likely that some degree of fine-tuning is required along the developmental trajectory, the understanding of which would allow a directed recapitulation of these mechanisms to obtain the respective stages^79^.

Although a knockdown of MORC alone was not sufficient to produce infectious sporozoites^84^, knockdown or the use of HDAC3 inhibitors are undoubtedly valuable instruments that could help in obtaining some sort of sexual stages *in vitro,* thereby providing a source for stage-specific RNA, protein or metabolites as the main objectives rather than the “real” sexual stage (Figure 1C).

### Use of intestinal organoids to mimic physiological conditions

Stem cell-derived organoids reflect, to a large extent, the cellular complexity of the tissues they are derived from and provide a potentially unlimited source for complex tissues like the intestine^90^. They were suggested early on as ’more complete’ *in vitro* host systems for many parasites, including *T. gondii*^91,92^. Their various cell constituents can be seeded as monolayers that display the polarization and electrophysiological properties of, for example, the intestinal epithelium, providing easy access to the apical surface for infection experiments with *T. gondii*^31,93^. This has also been achieved by generating collagen-supported epithelial sheets from intestinal organoids^94^. Immune cells and the microbiota are missing in these systems but can be added in co-culture approaches^95,96^. Several recent reviews have highlighted the great potential organoid-based systems will have as future cell culture models to either complement or, depending on the research question, even replace animal experiments in *T. gondii* and other Apicomplexa^97–100^.

In contrast to asexual replication, the sexual development of *T. gondii* is restricted to the feline intestinal epithelium. This fact has puzzled *T. gondii* researchers for decades. Experiments with cell lines derived from the feline intestinal epithelium have provided evidence that infection with bradyzoites could lead to the development of type C and D schizonts^101,102^. A big step forward to explain this remarkable species specificity, despite *T. gondii*’s otherwise promiscuous host range, was reported recently by Martorelli Di Genova *et al*. in 2019^6^. The authors explained this strict specificity with high levels of linoleic acid accumulating in the feline intestine due to a general lack of delta-6-desaturase (D6D) in this animal family, an enzyme responsible for catalyzing the reaction of linoleic acid to linolenic acid. The excess of linoleic acid enabled sexual development for reasons yet to be determined^6^. Infection of feline and murine intestinal organoid-derived cell cultures with bradyzoites resulted in the expression of pre-sexual stage marker proteins, although no infectious oocysts could be observed^6^. In contrast, when inhibiting the D6D enzyme in mice and feeding a linoleic acid-rich diet, sexual stages were detected in the intestinal epithelium (Figure 1C). Eventually, these mice shed oocyst-like structures with their feces that sporulated and were infectious, although the yield was not very high. The obtained oocysts showed reduced resistance to bleach and 2% sulfuric acid, compared to oocysts from cats, suggesting that additional factors are required for the formation of mature oocysts, like missing environmental (external) cues.

While intestinal organoids could be very promising tools for recapitulating *T. gondii* sexual stage development *in vitro,* the robustness of the method described by Martorelli Di Genova *et al*. in terms of, for example, organoid species or origin is currently unknown. Moreover, the reported yield of oocyst-like structures in cell culture was fairly low, and the use of a D6D inhibitor to suppress its activity in wildtype mice or organoids is both costly and potentially detrimental to the yield due to the possible side effects from the compound. Organoids from D6D knockout mice^103^ or from cats might thus be the better alternative. A potential pitfall for oocyst production lies in the fact that bradyzoites can revert to tachyzoites instead of entering the sexual developmental pathway (Figure 1C; Figure 2C, D). This could lead to host cell lysis and destabilization of the cell culture system and minimize oocyst yield. Two inhibitors of elF2α dephosphorylation, salubrinal (SAL) and guanabenz (GA), have been shown to prevent reactivation of bradyzoites and could be explored for the purpose of stabilizing such organoid cultures^5^.

Once robust oocysts-organoid culture systems have been developed, they may be used to screen the effect of various host factors, e.g., modified by gene knock-outs or pharmacological interventions, on sexual development. Likewise, the role of selected microbiota species could be studied (notably, germ-free cats have been reported to shed oocysts upon infection^104^). The *in vitro* system would also permit monitoring stage progression “as it happens”, using live microscopy and transgenic parasites tagged with different fluorescent markers for the respective intermediate stages^79^. While cultivating and handling of organoids is resource-intensive and typically done in small culture volumes, the development of new protocols for large-scale organoid expansion^105^ shows that this bottleneck can likely be overcome in the future. Also, mouse sarcoma-derived extracellular matrices required for organoid culture are not only expensive and in exceedingly short supply but are also ethically questionable. However, the large organoid research community is actively looking for synthetic alternatives (for review see 106). Taken together, as an unlimited source of primary intestinal cells, organoids could form the basis for an *in vitro* oocyst production system that could replace cats as a source of oocysts for research in the (not too distant) future.

## Concluding remarks

Although not all stages of the full life cycle of *T. gondii* have yet been recapitulated *in vitro*, considerable progress has been made in recent years in understanding its mechanisms and regulation. The tachyzoite stage can be readily cultivated, and new methods have been developed to obtain mature bradyzoites in very good yield that might already replace mouse-derived bradyzoites in many experiments. Combining all three stages into a single culture system, at least for analytical, microscopy-based experiments, will be challenging but not impossible, given the almost monthly advances in lab-on-a-chip approaches^95,107^.

Exploiting certain molecular factors like MORC or BFD1 to enable life cycle progression *in vitro* requires genetic manipulations of the parasite. In contrast, small molecules such as those described above can be added directly to the medium, also allowing the analysis of, for example, newly isolated *T. gondii* strains. Obviously, the ideal cell culture system would function without these makeshift measures to avoid any potentially undesirable effects on the parasite. Currently, however, they can be of great benefit when the main goal is the production of stage-specific biomolecules rather than infective stages.

The ambitious goal to fully recapitulate the sexual cycle of Coccidia has recently been achieved for *Cystoisospora suis*^108^ and *Cryptosporidium parvum*^109^, two relatives of *T. gondii*. The enteropathogen *C. suis* completes its entire developmental cycle in one host (swine). Interestingly, host cells were only required for asexual replication and until commitment of merozoites. Sexual development and fertilization readily took place in a cell-free culture system^108^, a finding supported by recent transcriptomic data^110^.

The life cycle development of *C. parvum*, which also takes place in a single host and requires less than three days, was modeled *in vitro* using organoid-derived “air-liquid interface” cultures. Remarkably, the system also supported genetic crosses and the generation of viable, recombinant oocysts^109^. In another recent study of *C. parvum,* live cell imaging was used to follow the entire life cycle in detail, including nuclear division of asexual and sexual stages of the parasite over multiple rounds of invasion and egress^111^. It would be fascinating to visualize and study the life cycle of *T. gondii* using *in vitro* models in a similar fashion. For *T. gondii*, it is still unclear where fertilization takes place. Since oocyst wall formation is observed inside the host cell, it is likely that microgametes enter the host cell to fuse with intracellular macrogametes^74,112^. Another interesting cell culture technology developed for *C. parvum* that could be useful for culturing other Apicomplexa are hollow fiber bioreactors^113^. The system provides a biphasic environment that can mimic the different oxygen and nutrient availabilities at the apical and basal surfaces of intestinal cells. The system was specifically designed for long-term culture and could be particularly valuable for increasing the number of parasites obtained.
